# *miR-146a* Polymorphism (rs2910164) Predicts Colorectal Cancer Patients’ Susceptibility to Liver Metastasis

**DOI:** 10.1371/journal.pone.0165912

**Published:** 2016-11-08

**Authors:** Tomohiro Iguchi, Sho Nambara, Takaaki Masuda, Hisateru Komatsu, Masami Ueda, Shinya Kidogami, Yushi Ogawa, Qingjiang Hu, Kuniaki Sato, Tomoko Saito, Hidenari Hirata, Shotaro Sakimura, Ryutaro Uchi, Naoki Hayashi, Shuhei Ito, Hidetoshi Eguchi, Keishi Sugimachi, Yoshihiko Maehara, Koshi Mimori

**Affiliations:** 1 Department of Surgery, Kyushu University Beppu Hospital, 4546 Tsurumihara, Beppu 874–0838, Japan; 2 Department of Surgery and Science Graduate School of Medical Sciences Kyushu University 3-1-1 Maidashi, Higashi-ku, Fukuoka 812–8582, Japan; University of South Alabama Mitchell Cancer Institute, UNITED STATES

## Abstract

*miR-146a* plays important roles in cancer as it directly targets *NUMB*, an inhibitor of Notch signaling. *miR-146a* is reportedly regulated by a G>C polymorphism (SNP; rs2910164). This polymorphism affects various cancers, including colorectal cancer (CRC). However, the clinical significance of *miR-146a* polymorphism in CRC remains unclear. A total of 59 patients with CRC were divided into 2 groups: a CC/CG genotype (n = 32) and a GG genotype (n = 27), based on the *miR-146a* polymorphism. cDNA microarray analysis was performed using 59 clinical samples. Significantly enriched gene sets in each genotype were extracted using GSEA. We also investigated the association between *miR-146a* polymorphism and *miR-146a*, NUMB expression or migratory response in CRC cell lines. The CC/CG genotype was associated with significantly more synchronous liver metastasis (p = 0.007). A heat map of the two genotypes showed that the expression profiles were clearly stratified. GSEA indicated that Notch signaling and JAK/STAT3 signaling were significantly associated with the CC/CG genotype (p = 0.004 and p = 0.023, respectively). CRC cell lines with the pre-*miR-146a*/C revealed significantly higher *miR-146a* expression (p = 0.034) and higher NUMB expression and chemotactic activity. In CRC, *miR-146a* polymorphism is involved in liver metastasis. Identification of this polymorphism could be useful to identify patients with a high risk of liver metastasis in CRC.

## Introduction

Colorectal cancer (CRC) is the third most common neoplasm worldwide and is the second leading cause of cancer-related deaths in developed countries, with the majority of deaths attributable to distant metastasis [1,2]. Despite major advancements in diagnostic and therapeutic approaches to CRC, the prognosis of patients with distant metastasis commencing with liver metastasis is still unfavorable [3,4]. Therefore, there is an urgent need to establish a novel biomarker for cancer progression and metastasis in CRC.

MicroRNAs (miRNAs) are non-coding RNAs with lengths between 21 to 25 nucleotides. They bind the 3’-untranslated region (UTR) of various target mRNAs, enhancing their degradation or their translational repression, leading to multiple biological consequences [5,6]. In several cancers, *miR-146a* (encoded on chromosome 5q33) is dysregulated and acts as an oncogene [7–9] or as a tumor-suppressor gene [10–14]. Meanwhile, Snail, an inducer of the epithelial to mesenchymal transition, induces *miR-146a*, stabilizing Wnt activity and attenuating tumorigenicity [15]. Moreover, higher levels of *miR-146a* expression in CRC are associated with longer survival, suggesting it acts as a tumor-suppressor gene [16].

Single nucleotide polymorphisms (SNPs) in miRNA have attracted attention because the SNPs may affect the expression and function of the miRNAs and be involved with the initiation and progression of cancer [17–19]. A common genetic variant rs2910164 located within the *miR-146a* precursor sequence was reported to change the stability of pri-miR by mispairing within the hairpin, followed by a reduction in the predicted ΔG from -43.1 kcal/mol to -40.3 kcal/mol [20]. The association between *miR-146a* polymorphism and the susceptibility to CRC has been well studied [21–24].With regard to the progression of CRC, Chae et al. demonstrated that patients with pre-*miR-146a*/C have a poor outcome [24], however, little is known about the clinicopathological relevance of *miR-146a* polymorphism in CRC. Here, we show that the association between the *miR-146a* polymorphism and liver metastasis in CRC occurs through Notch signaling and JAK/STAT3 signaling.

## Materials and Methods

### Collection of samples from CRC patients

Between December 2005 and July 2006, 59 CRC patients who underwent surgery at the National Cancer Center Hospital were enrolled in this study. This project was approved by Kyushu University Institutional Review Board for Human Genome/Gene Research and written informed consent was obtained from each patient. We collected peripheral blood mononuclear cell samples to determine the genotype of the *miR-146a* polymorphism. Resected tumor samples were immediately cut and stored in RNA*later* (Ambion) or embedded in Tissue-Tek OCT (optimum cutting temperature) medium (Sakura, Tokyo, Japan), frozen in liquid nitrogen and kept at -80°C until RNA extraction.

### CRC cell lines

Human CRC cells (RKO, HT29, WiDr, CaR1, LoVo, COLO320 and DLD1) were provided by the Japanese Cancer Research Bank (Tokyo, Japan). Cell lines were maintained in Dulbecco’s Modified Eagle’s medium supplemented with 10% fetal bovine serum and antibiotics. All cells were cultured as monolayers at 37°C in a humidified atmosphere containing 5% CO_2_.

### Total RNA extraction

Tumor samples were used as pure cancer cells separated by laser microdissection as described in our previous report [25]. Total RNA from clinical samples and CRC cell lines were extracted using the modified acid-guanidine-phenol-chloroform method [26].

### Genomic DNA extraction

Genomic DNA was extracted from peripheral blood samples from 59 cases of CRC by means of conventional methodologies, then quantified with PicoGreen (Invitrogen, Carlsbad, CA).

### Quantitative reverse transcription PCR (RT-qPCR)

Quantitative analysis of *miR-146a* and *RNU6B* (internal control) was performed using specific cDNAs derived from total RNA extracted from CRC cell lines using gene-specific primers, according to the TaqMan MicroRNA Assay protocol (Assay IDs: 0000449 for has-*miR-146a* and 001093 for *RNU6B*; Applied Biosystems, Carlsbad, California, USA), as previously described [27]. The raw miR expression levels were normalized to *RNU6B* expression for calculation of the relative miR expression values.

RT was performed using an M-MLV reverse transcriptase kit according to the manufacturer’s protocol (Invitrogen). cDNA was generated from 8 μg total RNA in a 30 μL reaction mixture and was diluted up to 100 μL with TE. To determine the relative expression levels of *NUMB*, qPCR was performed with a LightCycler 480 instrument (Roche Applied Science, Basel, Switzerland) using a LightCycler 480 Probes Master kit (Roche Applied Science), according to the manufacturer’s protocols. The sequences of the primers for *NUMB* were as follows: sense, 5’-GTTGTCATGGGGGAGGTG-3’ and antisense, 5’-TTGCTTAAGCCTCAAATCTGC-3’. Glyceraldehyde-3-phosphate dehydrogenase (*GAPDH*) primers were as follows: sense, 5’-TTGGTATCGTGGAAGGACTCTCA-3’ and antisense, 5’-TGTCATATTTGGCAGGTT-3’; *GAPDH* served as the internal control to normalize the expression level of *NUMB*. The amplification conditions were as follows: 10 min at 95°C followed by 45 cycles of 10 s at 95°C and 30 s at 60°C. The expression levels were expressed as the values relative to the expression levels of Human Universal Reference Total RNA (Clontech, Palo Alto, CA, USA).

### Immunoblotting analysis

Total protein was extracted from CRC cell lines with RIPA buffer. Immunoblotting was performed as described in our previous report [28]. In brief, NUMB protein was detected using rabbit polycolonal antibodies (ab14140; Abcam)?. Protein levels were normalized to the level of β-actin protein, which was detected using mouse monoclonal antibodies (Cytoskeleton, Denver, CO, USA). Blots were developed with horseradish peroxidase-linked anti-rabbit or anti-mouse immunoglobulin (Promega, Madison, WI, USA). Enhanced chemiluminescence detection reagents (Amersham Biosciences, Piscataway, NJ, USA) were used to detect antigen-antibody reactions.

### Transfection of miR-146a inhibitor

Either miR-146a inhibitor or its negative control (miRVana® miRNA inhibitor, Life technologies, MH10722, hsa-miR-146a-5p) was transfected into CRC cell lines with the pre-*miR-146a*/C (Lovo and DLD-1) using Lipofectamine RNAiMAX Transfection Reagent (Thermo Fisher Scientific) according to manufacture’s protocols. Then, total RNA and protein were extracted for further experiments.

### Migration assay

Cell migration was assessed using the BD Falcon FluoroBlok 24 Multiwell Insert System (BD Bioscience, San Jose, CA). The cells (1.0x10^5^ cells/500 μl/well) were then placed in the upper chamber of the 24-well plate with serum-free medium. The lower chamber was filled with 10% fetal bovine serum, which acts as a chemoattractant, and incubated in a humidified atmosphere (37°C and 5% CO_2_). After a 48 h incubation, invasive cells that migrated through the membrane were evaluated in a fluorescence plate reader at excition /emission wavelengths of 485/530 nm. Invasiveness was measured as the percentage of fluorescence of an invasive fibrosarcoma cell line (HT-1080) that served as a control. Each independent experiment was performed with at least three replicates.

### PCR Amplification of Markers and DNA direct sequencing

The TaqMan probes and primers for rs2910164 were purchased from Applied Biosystems (assay ID C_15946974_10). Genotyping of clinical samples was performed with the ABI 7900HT Sequence Detection System and SDS 2.0 software (Applied Biosystems). In addition, *miR-146a* polymorphism of CRC cell lines was analyzed by PCR direct sequencing as described in a previous report [29,30]. The sequences of primers for a 192 bp fragment containing *miR-146a* polymorphism site (rs2910164) were as follows: 5’-CCGATGTGTATCCTCAGCTTTG-3’ and 5’-GCCTGAGACTCTGCCTTCTG-3’.

### Expression Analysis by cDNA Microarray

We performed microarray assays on 59 CRC tissues with the Human Whole Genome Oligo DNA Microarray Kit (Agilent Technologies, Santa Clara, CA) as described in our previous report [25]. A list of expressed genes on this cDNA microarray is available online (http://www.chem.agilent.com/scripts/generic.asp?1page=5175&indcol=Y&prodcol=Y&prodcol=N&indcol=Y&prodcol=N). A hierarchical cluster analysis of all the samples was performed by Euclidean distance and Ward’s linkage algorithms.

### Gene Set Enrichment Analysis (GSEA)

The association between *miR-146a* polymorphism and previously annotated gene expression signatures was analyzed by applying GSEA using expression profiles from cDNA microarray analysis of 59 CRC patients whose *miR-146a* polymorphism was provided, as previously described [28]. Gene sets extracted from the Broad Institute database and the Uniform Resource Locator of their source are as follows: KEGG_NOTCH_SIGNALING_PATHWAY (http://software.broadinstitute.org/gsea/msigdb/cards/KEGG_NOTCH_SIGNALING_PATHWAY) and V$STAT3_01 (http://software.broadinstitute.org/gsea/msigdb/cards/V$STAT3_01).

### Statistical analysis

χ^2^ tests or Fisher’s exact test was used for comparisons between *miR-146a* polymorphisms and clinicopathological factors. Comparisons of *miR-146a*, *NUMB* expression or migratory response between a dominant model of *miR-146a* polymorphism were evaluated using Mann-Whitney’s U-test. These results were analyzed using JMP 9 software (SAS Institute, Cary, NC, USA) or R version 3.1.1 (R Core Team (2014). R: A language and environment for statistical computing. R Foundation for Statistical Computing, Vienna, Austria. URL: http://www.R-project.org/). P values less than 0.05 were considered statistically significant.

## Results

### Correlations between the *miR-146a* polymorphism and clinicopathological factors

We divided the 59 patients with CRC into 2 groups: those with the CC/CG genotype, having the pre-*miR-146a*/C (n = 32) and the GG genotype, not having pre-*miR-146a*/C, according to the dominant model of *miR-146a* polymorphism and compared the clinicopathological findings between the two genotypes (Table 1). Intriguingly, whereas no significant differences were noted with respect to distant metastasis, peritoneal dissemination or other factors pertaining to tumor progression, liver metastasis occurred more frequently in the CC/CG genotype than the GG genotype (p = 0.007).

**Table 1:** Comparative analysis of the clinicopathological findings affected by miR-146a polymorphism.

Factor	GG genotype(n = 27)	CC/CG genotype(n = 32)	p-value
Age (years)*	62.6 ± 9.7	60.3 ± 11.3	0.407
Sex (male/female)	13/14	17/15	0.703
Location (right side/left side/rectum)	7/9/11	12/8/12	0.606
Tumor size (cm)*	4.8 ± 1.6	5.6 ± 1.8	0.064
Histological differentiation (Well/Mode)	16/11	26/6	0.063
T factor (T1, T2/T3, T4)	5/22	4/28	0.522
Lymphatic invasion (%)	22.2	31.3	0.437
Venous invasion (%)	48.1	62.5	0.269
Lymph node metastasis (%)	59.3	65.6	0.614
Distant metastasis (%)	3.7	12.5	0.227
Liver metastasis (%)	3.7	31.3	0.007
Peritoneal dissemination (%)	0.0	6.3	0.186
Stage, UICC 7th (I/II/III/IV)	4/6/16/1	2/8/12/10	0.036

Abbreviations: Well, well differentiated adenocarcinoma; Mode, moderately differentiated adenocarcinoma; UICC, Union for international cancer control

*average ± standard deviation

### Different cDNA expression profiles and gene set signatures found in the *miR-146a* polymorphism

**Fig 1:**
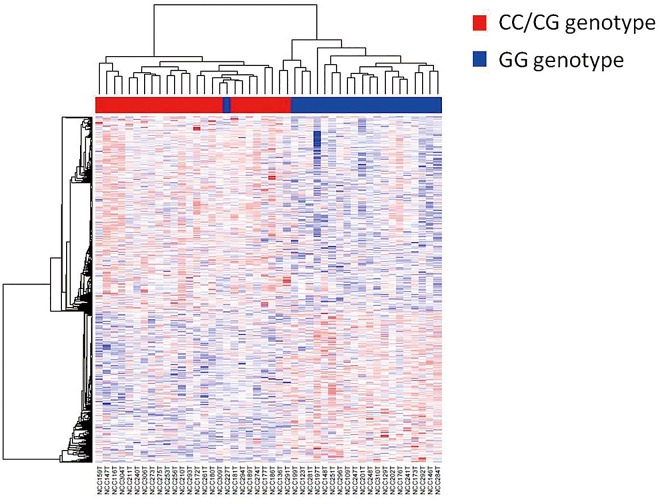
Hierarchical clustering showing the expression levels of the differentially expressed genes in a comparison of CRC patients with the pre-*miR-146a*/C (CC/CG) genotype and without the pre-*miR-146a*/C (GG) genotype. Red spots indicate upregulated and blue spots indicate downregulated probes compared with reference probes. On the top, clustering results of CRC patients are shown (dendrogram). Red bar indicates the CC/CG genotype and the blue bar indicates the GG genotype. On the left side, clustering results of the differentially expressed genes between the two genotypes are shown.

**Fig 2:**
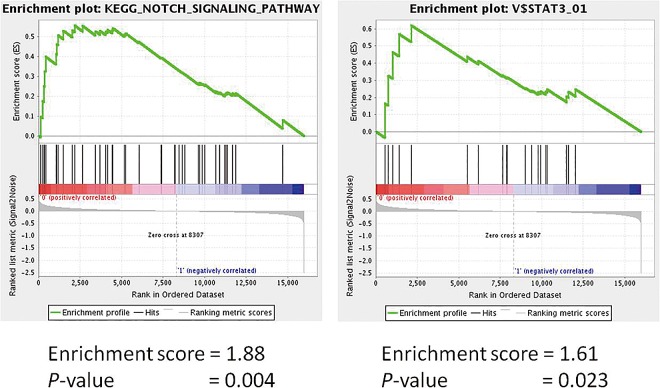
Gene Set Enrichment Analysis (GSEA): Enriched gene sets for CRC patients with the C allele (CC/CG); KEGG_NOTCH_SIGNALING_PATHWAY (A) and V$STAT3_01 (B).

From the microarray assay, we identified 570 significantly upregulated genes and 443 significantly downregulated genes in the CC/CG genotype compared with the GG genotype. We examined a heat map of the clustering of the expression pattern of those genes in the 59 CRC cases (Fig 1). The characteristics of the expression profiles of the *miR-146a* polymorphism were clearly stratified. Furthermore, GSEA indicated that a number of Notch signaling pathway signatures and JAK/STAT3 signaling pathway signatures were significantly enriched in the CC/CG genotype (p = 0.004 and p = 0.023, respectively) (Fig 2).

### The influence of the *miR-146a* polymorphism on the expression of *miR-146a* and NUMB and migratory response

**Fig 3:**
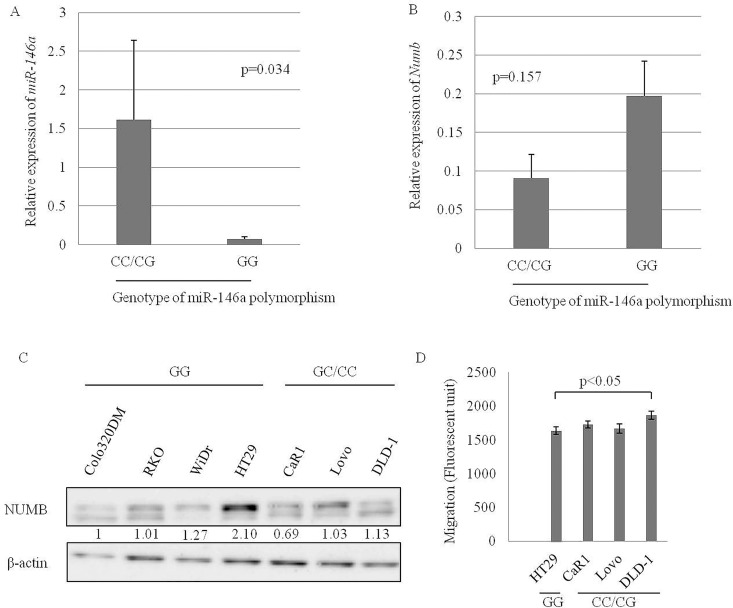
Comparison of *miR-146a* expression (A) and *NUMB* expression (B) between CRC cell lines with and without the pre-*miR-146a*/C. NUMB expression of CRC cell lines by immunoblotting (C). Migratory capacity of CRC cell lines with the pre-miR-146a/C compared with HT29, a representative CRC cell line without pre-miR-146a/C (D).

To explore the influence of *miR-146a* polymorphism on the expression of *miR-146a* and *NUMB*, we examined the relationship between the polymorphism and the expression of *miR-146a* or *NUMB* in 7 CRC cell lines, whose polymorphism was determined by direct sequencing (Table 2). *miR-146a* expression was significantly higher in 3 CRC cell lines with the pre-*miR-146a*/C than the 4 CRC cell lines without the pre-*miR-146a*/C (p = 0.034) (Fig 3A). There was no association between the *miR-146a* polymorphism and *NUMB* expression in the CRC cell lines (Fig 3B), however, immunoblotting analysis revealed that NUMB expression of CRC cell lines with pre-miR-146a/C (CaR1, Lovo and DLD-1) was lower than WiDr and HT29, CRC cell lines without pre-miR-146a/C (Fig 3C). Furthermore, DLD-1, one CRC cell line with the pre-*miR-146a*/C had higher chemotactic activity than the CRC cell lines without the pre-*miR-146a*/C (Fig 3D).

**Table 2:** Genotyping of miR-146a polymorphism in 7 CRC cell lines.

CRC cell lines	Genotype of rs2910164
RKO	GG
HT29	GG
WiDr	GG
CaR1	CC
LoVo	GC
COLO320	GG
DLD1	CC

Abbreviations: CRC, colorectal cancer

### Inhibition of *miR-146a* expression increased NUMB expression in CRC

**Fig 4:**
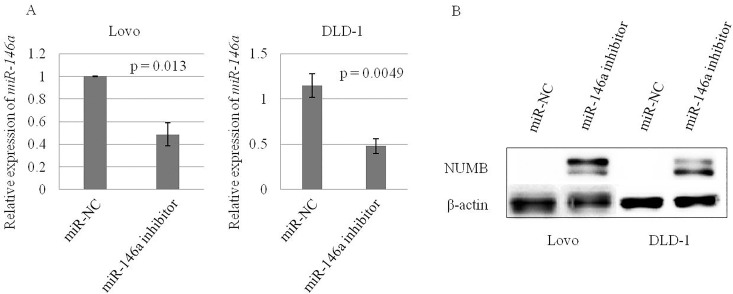
Downregulated *miR-146a* (A) and upregulated NUMB (B) expression of CRC cell lines with pre-miR-146a/C transfected with miR-146a inhibitor or negative control.

In order to confirm the influence of *miR-146a* expression on the NUMB expression in CRC, we investigated NUMB expression of CRC cell lines with the pre-*miR-146a*/C treated by miR-146a inhibitor. Knockdown of miR-146a, confirmed by RT-qPCR (Fig 4A) enhanced NUMB expression in CRC cell lines with the pre-*miR-146a*/C (Fig 4B).

## Discussion

Numerous previous studies have described the role of *miR-146a* polymorphism in patients’ susceptibility to CRC. In this study, we demonstrated that *miR-146a* polymorphism was also involved with liver metastasis in CRC patients. We hypothesized that the molecular mechanism of liver metastasis in CRC is caused by dramatic changes of gene signatures affected by *miR-146a* polymorphism. GSEA showed that Notch signaling and JAK-STAT3 signaling were significantly activated in the patients with pre-*miR-146a*/C. Notch signaling may be involved in cell-fate decision and promote cell survival or anti-apoptosis by mediating the expression of some anti-apoptosis or pro-survival proteins [31,32]. Notch signaling also mediates the epithelial-mesenchymal transition process, leading to metastasis [31] and has been traditionally implicated as a key mechanism for the initiation and progression in CRC [33]. Sonoshita et al. demonstrated that inhibition of Notch signaling by amino-terminal enhancer of split (Aes) suppressed the invasiveness and the intravasation of CRC cells in an orthotopic transplantation model [34]. We previously reported that Notch induced CCL2 which in turn promoted distant metastasis in vivo [35]_._ Another pathway HGF/c-MET/ETS-1 also plays a central role in tumor proliferation, invasion and metastasis [36,37]. However, GSEA showed no significant enrichment for the gene set of HGF/c-MET/ETS-1 pathway (data not shown). Thus, *miR-146a* polymorphism may promote liver metastasis in CRC via Notch signaling and JAK/STAT3 signaling.

It is well known that the common genetic variant rs2910164 is located within the *miR-146a* precursor sequence and that it affects the expression of mature *miR-146a* [29,30,38–41]. The pre-*miR-146a*/C genotype may reduce the stability of pri-miR by mispairing events within the hairpin [42] and it has been associated with lower expression of the *miR-146a* in several cancers [38–41]. We previously reported that patients with the pre-*miR-146a*/C genotype showed higher expression of *miR-146a* than those with the pre-*miR-146a*/G genotype in gastric cancer [30]. The effect of *miR-146a* polymorphism on the expression of *miR-146a* has been demonstrated *in vitro* [29]. In our study, the clinical samples whose SNP was examined were unfortunately unavailable. However, CRC cell lines with the pre-*miR-146a*/C genotype have significantly higher *miR-146a* expression than those with the pre-*miR-146a*/G. This discrepancy may be explained by the ethnicity, cancer type, and disease status.

NUMB protein is a key negative regulator of Notch signaling [43] and is one of the direct targets of *miR-146a* [15,41,44,45]. *miR-146a* induces an oncogenic phenotype and tumorigenesis of oral squamous cell carcinoma by directly targeting the 3’-UTR of *NUMB* [45]. In CRC, Snail induces *miR-146a* expression, which in turn targets *NUMB* and stabilizes β-catenin [15]. Contrary to our result with *miR-146a* expression, CRC cell lines with the pre-*miR-146a*/C genotype had the lower expression of NUMB protein and more migratory response than those with a pre-*miR-146a*/G genotype. NUMB suppression may be affected by alteration of *miR-146a* expression through this SNP, followed by increasing the migratory response via enhancing Notch signaling; however, further examination is needed to confirm the effect of *miR-146a* polymorphism on liver metastasis in CRC using genome editing.

In conclusion, we demonstrated that *miR-146a* polymorphism is associated with the tendency of CRC to metastasize to the liver. This occurs through *miR-146a-*mediated activation of Notch and JAK/STAT3 signaling via suppression of NUMB. Identification of *miR-146a* polymorphism could identify patients with high-grade CRC and be useful for follow-up with particular attention to liver metastasis.
